# Sources of coronavirus disease 2019 (COVID-19) exposure among healthcare personnel (HCP) in a large tertiary-care medical center

**DOI:** 10.1017/ash.2023.157

**Published:** 2023-04-28

**Authors:** Jana Shaw, Paul Suits, Heidi Steigerwald, Stephen J. Thomas, Margaret K. Formica

**Affiliations:** 1 Division of Infectious Diseases, Department of Pediatrics, Upstate Golisano Children’s Hospital, SUNY Upstate Medical University, Syracuse, New York; 2 Department of Infection Prevention, Upstate Medical University, Syracuse, New York; 3 Marcellus High School, Marcellus, New York; 4 Department of Internal Medicine, Institute for Global Health and Translational Science, SUNY Upstate Medical University, Syracuse, New York; 5 Department of Public Health and Preventive Medicine and Department of Urology, SUNY Upstate Medical University, Syracuse, New York [Present affiliation: University of Connecticut, Storrs, Connecticut (H.S.).]

## Abstract

**Objectives:**

To describe the burden and sources of severe acute respiratory coronavirus virus 2 (SARS-CoV-2) infection among healthcare personnel (HCP), such as occupational role, work setting, vaccination status, and patient contact between March 2020 through May 2022.

**Design:**

Active prospective surveillance.

**Setting:**

Large tertiary-care teaching institution with inpatient and ambulatory care services.

**Results:**

We identified 4,430 cases among HCPs between March 1, 2020, through May 31, 2022. The median age of this cohort was 37 years (range, 18–89); 2,840 (64.1%) were female; and 2,907 (65.6%) were white. Most of the infected HCP were in the general medicine department, followed by ancillary departments and support staff. Less than 10% of HCP SARS-CoV-2–positive cases worked on a COVID-19 unit. Of the reported SARS-CoV-2 exposures, 2,571 (58.0%) were from an unknown source, 1,185 (26.8%) were from a household source, 458 (10.3%) were from a community source, and 211 (4.8%) were healthcare exposures. A higher proportion of cases with reported healthcare exposures was vaccinated with only 1 or 2 doses, whereas a higher proportion of cases with reported household exposure was vaccinated and boosted, and a higher proportion of community cases with reported and unknown exposures were unvaccinated (*P* < .0001). HCP exposure to SARS-CoV-2 correlated with community-level transmission regardless of type of reported exposure.

**Conclusions:**

The healthcare setting was not an important source of perceived COVID-19 exposure among our HCPs. Most HCPs were not able to definitively identify the source of their COVID-19, followed by suspected household and community exposures. HCP with community or unknown exposure were more likely to be unvaccinated.

Healthcare personnel (HCP) are considered to be at greater risk for occupational exposure to severe acute respiratory coronavirus virus 2 (SARS-CoV-2) infection due to their prolonged and frequent exposure to infected patients and unprotected contacts with undiagnosed patients, coworkers, and visitors.^1–6^ In the early stages of the pandemic prior to the availability of vaccines, the risk of exposure in healthcare setting was high. HCP accounted for 8.8%–13.8% of new COVID-19 cases, according to reports published prior to COVID-19 vaccine availability.^7–9^

With the advent of vaccines, universal testing of hospitalized patients, those undergoing elective procedures, restrictive patient visiting policies, and improved personal protective equipment (PPE) supply, the incidence of occupational infections was expected to decline. Multiple waves of high community SARS-COV-2 spread due to the emergence of more contagious variants and decreased vaccine effectiveness against infection with new variants, raised questions about other sources of infection for HCPs. A recent retrospective cohort study showed that the highest sources of infections were unknown (53.1%), followed by the household (27.1%) and community (15.6%). Occupational exposures accounted for 3.6% of HCP infections. Unattributable and community-acquired infections correlated closely with the community level of SARS-CoV-2 transmission.^10^

Furthermore, occupational factors such as role, setting, and PPE availability have been associated with seroprevalence rates in HCPs. However, it remains unsettled whether these occupational features translate into higher risk of infection among HCPs relative to the risk of community transmission, and if so, which HCP are at highest risk.^4,8,9,11^

To address the gap, we conducted active prospective surveillance for all employed HCP with newly diagnosed with COVID-19 between March 1, 2020, and May 31, 2022. We inquired about their sources of exposure using a standardized health department checklist and Centers for Disease Control and Prevention (CDC) guidance for managing HCP with SARS-CoV-2 infection or exposure. We describe the burden of SARS-CoV-2 infection among HCP in a large healthcare facility, sources of infection, and occupational factors such as role, setting, vaccination status, and patient contact.

## Methods

### Study population setting

SUNY Upstate Medical University in Syracuse, New York, is the only academic medical center in Central New York, and it is the region’s largest employer, with >9,000 employees.

### Data collection and management

We conducted active surveillance of all individuals who worked at the medical center, either as institutional employees, external contractors, volunteers, or learners who were newly diagnosed with COVID-19 between March 1, 2020, and May 31, 2022. Upon diagnosis, we inquired about their sources of exposure using a standardized health department checklist and CDC guidance for managing HCP with SARS-CoV-2 infection or exposure.^12^ HCP participated in public health guidelines for screening prior to entry in a healthcare facility by attesting to having no symptoms of COVID-19, or positive SARS-CoV-2 test, or close contact with someone infected with COVID-19. Attestation was provided by scanning their employee badge at the work entrance using a kiosk, using a “chatbot” to solicit symptoms, or in our ambulatory setting, staff had their temperature taken and attested to no symptoms on paper. If staff used the “chatbot” and identified symptoms, the application guided them to register for testing at one of our many locations. Any staff person who developed symptoms at work was instructed by management to call the employee hotline to register for testing at one of our convenient locations. All SARS-CoV-2–positive staff members populated a laboratory-generated SARS report that populated the infection prevention Epic “Bugsy” dashboard daily (Epic Systems, Verona, WI). Additionally, any staff member who tested positive outside the institution was instructed to call the employee student health office or infection prevention department for an exposure interview.

Contract tracers used the daily “Bugsy” report and called all HCP with positive test results to fill out a questionnaire. Results were entered into a self-serve application to track the key information over the course of the pandemic. This information included name, demographics, employee title, department, title, work location, vaccination status, test date, symptom onset date, a list of symptoms, last date worked, unmasked contact, and the type of exposure identified (healthcare-patient or coworker, community, household or unknown). We followed the isolation and quarantine guidelines provided by the CDC,^13^ and any additional guidelines offered by the New York State Department of Health (NYSDOH).^14^

The dates of COVID-19 cases were determined based on the date of a positive SARS-CoV-2 test (PCR or antigen based) or when unavailable, the date the case was reported to the institution. Employment records were used to determine the department of employment, role in the institution, level of patient care (direct, indirect, or none), COVID-19–unit exposure, and vaccination status and vaccination dates. Information on COVID-19 exposure and whether contact was masked or unmasked was obtained from the health department checklist.

Daily counts of SARS-CoV-2–positive cases for the Central New York region during the same period were obtained from the New York State Department of Health, New York State Statewide COVID-19 testing data, which were downloaded on July 29, 2022.^15^

A first dose of COVID-19 vaccine was required for hospital and long-term facility workers by September 27, 2021.^16^ Two doses of COVID-19 vaccine were required for healthcare workers in New York State. NYSDOH issued a notice not to enforce a COVID-19 vaccine booster requirement due to concerns over staffing shortages on February 18, 2022.^17^ Vaccination status at the time of infection was recorded.

The SUNY Upstate Institutional Review Board determined that this project did not meet the definition of human-subject research under the purview of the institutional review board.

### Statistical analysis

Frequencies and percentages of employment characteristics, masked contact, and vaccination status were described across COVID-19 exposure categories and were then statistically compared using χ^2^ statistics. Cumulative incidences of COVID-19 were calculated for all cases and separately among each exposure type. Cumulative incidences overall, by role at the institution, and by level of patient contact were calculated by dividing the number of cases in each category by the total number of HCP employed by the institution within each category. We used χ^2^ statistics to compare cumulative incidences across institution role and level of patient contact. All statistical analyses were conducted using SAS version 9.4 software (SAS Institute, Cary, NC).

## Results

### Participant characteristics

Active surveillance of COVID-19 identified 4,430 SARS-CoV-2–positive HCP, including 516 repeat infections (11.2%). Their demographic and clinical characteristics are described in Table 1. Most infected HCP worked in the general medicine department, followed by ancillary departments and support staff. Less than 10% of SARS-CoV-2–positive HCP worked on a COVID-19 unit. Only 194 (4.4%) admitted unmasked contact with a patient or coworker, and 32.9% were fully vaccinated and boosted at the time of their infection.

**Table 1.** Characteristics of All Healthcare Personnel (HCP) COVID-19 Cases by Exposure (N=4,430)

	HCP COVID-19 Cases	
Characteristic	No.	%
**Age group**		
1–29 y	1,222	27.6
30–39 y	1,321	29.8
40–49 y	847	19.1
50–59 y	720	16.3
≥60 y	317	7.2
**Sex**		
Female	2,840	64.1
Male	925	20.9
Unknown	665	15.0
**Race**		
White	2,907	65.6
Black	424	9.6
Asian	236	5.3
Other	178	4.0
Unknown	685	15.5
**Department category**		
Ancillary^a^	551	12.4
Ambulatory	72	1.6
Intensive care unit	220	5.0
Emergency room	184	4.2
General	2,142	48.4
Hematology-oncology	94	2.1
Laboratory	144	3.3
Student	193	4.4
Support staff^b^	470	10.6
Surgery	360	8.1
**COVID-19 unit**		
Yes	413	9.3
No	4,017	90.7
**Unmasked contact**		
Yes	194	4.4
No	2,974	67.1
Unknown	1,262	28.5
**COVID-19 vaccine doses**		
No doses	1,548	34.9
1 or 2 doses	1,425	32.2
3 doses	1,457	32.9

a Ancillary services included clerical, dietary, phlebotomy, unit support, registration, clinical support, environmental staff.

b Support services included laboratory, informational technology, and pharmacy staff.

### Sources of SARS-CoV-2 exposure

Of the reported SARS-CoV-2 exposures, 2,571 (58.0%) were from an unknown source, 1,185 (26.8%) were from a household source, 458 (10.3%) were from a community source, and 211 (4.8%) were healthcare exposures (Table 2). A higher proportion of cases with reported healthcare exposures was vaccinated with only 1 or 2 doses, whereas a higher proportion of cases with reported household exposure was fully vaccinated and boosted, and a higher proportion of community cases with reported and unknown exposures was unvaccinated (*P* < .0001).

**Table 2.** Vaccine Status of All Healthcare Personnel (HCP) COVID-19 Cases by Exposure (N=4430)^a^

Characteristic	Healthcare Exposure (N=211), No. (%)	Household Exposure (N=1,185), No. (%)	Community Exposure (N=458), No. (%)	Unknown Exposure (N=2,571), No. (%)	*P* Value
**COVID-19 vaccine doses**					
No Doses	58 (27.5)	321 (27.1)	174 (38.0)	991 (38.6)	<.0001
1 or 2 Doses	90 (42.7)	396 (33.4)	154 (33.6)	784 (30.5)	
3 Doses	63 (29.9)	468 (39.5)	130 (28.4)	796 (31.0)	

a 5 cases (0.1%) were missing information regarding exposure type.

### Incidence of COVID-19 among HCP

Although active surveillance captured COVID-19 among all HCP during the study period, not all were directly employed by the institution (ie, contractors). Of the 4,430 identified cases, 3,088 (69.7%) were employees of the institution and were included in incidence calculations. Compared to the employees, the excluded nonemployees were younger, more likely to be students, and were less likely to be vaccinated. Table 3 displays cumulative incidences of COVID-19 among employee HCP overall, by hospital role, and by level of patient contact. The cumulative incidence of COVID-19 among the 8,766 HCP employees during the study period was 35.2%. There was statistically significant variability in incidence by hospital role (*P* < .0001), with registered nurses being the most likely to be infected (n = 888, 47.9%), followed by master’s level clinicians (n = 84, 39.6%). Administration and management were the least likely to be infected with SARS-CoV-2 (n = 173, 21.4%). Statistically significant variability was also found by level of patient contact (*P* < .0001), with those providing direct patient care having the highest incidence of COVID-19. Unknown exposure had the highest incidence (19.9%), followed by household exposure (10.0%), community exposure (3.6%), and healthcare exposure (1.8%). Statistically significant variability in COVID-19 incidence by hospital role and level of patient contact was also detected when examined by exposure type. Registered nurses with unknown exposure had the highest incidence by hospital role (26.4%). For level of patient care, HCP with unknown exposure and direct patient care had the highest incidence (22.6%).

**Table 3.** Incidence of COVID-19 Among Healthcare Personnel (HCP) Overall and by Exposure Type^a^

Characteristic	Total No. (% of Employees)	All Cases, No. (%)	Healthcare Exposure Cases, No. (%)	Household Exposure Cases, No. (%)	Community Exposure Cases, No. (%)	Unknown Exposure Cases, No. (%)
Overall	8,766 (100.0)	3,088 (35.2)	156 (1.8)	874 (10.0)	315 (3.6)	1,742 (19.9)
**Role category**						
Registered nurses	1,853 (21.1)	888 (47.9)	49 (2.6)	262 (14.1)	87 (4.7)	490 (26.4)
Scientists and physicians^b^	1,724 (19.7)	431 (25.0)	33 (1.9)	123 (7.1)	37 (2.2)	238 (13.8)
Administration and management^c^	810 (9.2)	173 (21.4)	2 (0.3)	49 (6.1)	16 (2.0)	105 (13.0)
Ancillary services^d^	2601 (29.7)	973 (37.4)	42 (1.6)	256 (9.8)	106 (4.1)	569 (21.9)
Technical staff^e^	764 (8.7)	228 (29.8)	3 (0.4)	72 (9.4)	31 (4.1)	122 (16.0)
Allied health professionals^f^	760 (8.7)	295 (38.8)	22 (2.9)	83 (10.9)	30 (4.0)	160 (21.1)
Master’s level clinicians^g^	212 (2.4)	84 (39.6)	2 (0.9)	27 (12.7)	8 (3.8)	47 (22.2)
Public safety and spiritual care	42 (0.5)	16 (38.1)	3 (7.1)	2 (4.8)	0 (0.0)	11 (26.2)
*P* value		<.0001	<.0001	<.0001	.0003	<.0001
**Patient contact**						
Direct	5,746 (65.6)	2,262 (39.4)	125 (2.2)	629 (11.0)	209 (3.6)	1298 (22.6)
Indirect	1,105 (12.6)	389 (35.2)	20 (1.8)	103 (9.3)	50 (4.5)	216 (19.6)
None	1,915 (21.9)	437 (22.8)	11 (0.6)	142 (7.4)	56 (2.9)	228 (11.9)
*P* value		<.0001	<.0001	<.0001	.0716	<.0001

a Information on exposure type is missing for 1 case.

b Includes research staff and medical students.

c Includes educational support and other.

d Includes clerical, dietary, phlebotomy, unit support, registration, clinical support, environmental services.

e Includes laboratory, informational technology, and pharmacy services.

f Includes physical therapy, occupational therapy, radiology, and respiratory services.

g Includes nurse practitioners, physician’s assistants, social workers, and registered dieticians.

### COVID-19 infection among HCP and community-level transmission

**Figure 1.**
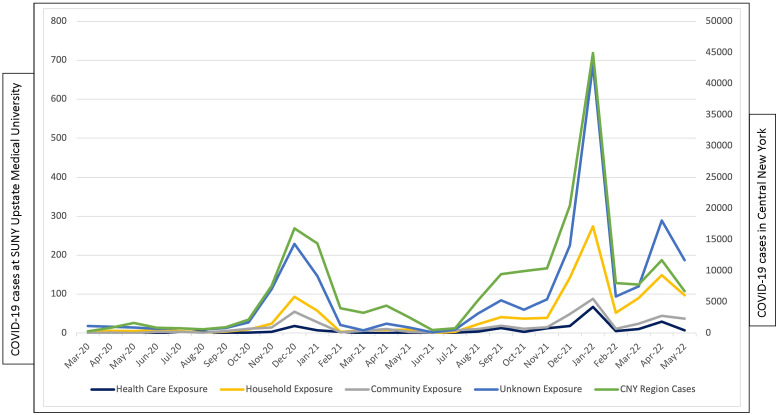
SUNY Upstate Medical University Health Care Personnel COVID-19 cases by source of exposure in the Central New York region between March 1, 2020, through May 31, 2022.

HCP exposure to COVID-19 correlated with community-level transmission regardless of type of reported exposure (Fig. 1). The sources of exposure did not change substantially over time (data not shown).

## Discussion

The majority of HCP had an unknown source of COVID-19 exposure, followed by household and community exposures. Healthcare setting was not an important source of infection—it was reported in <5% of HCP. Our findings are consistent with a recent report about sources of COVID-19 among HCP in Mayo Clinic, Rochester, Minnesota, where unattributable infections (no known exposure source) accounted for 53.1%, followed by household (27.1%) and community (15.6%) exposures. Occupational exposures accounted for 3.55% of HCP infections.^10^ Unattributable infections also correlated with community rather than occupational exposure. These findings suggest that infection prevention policies at healthcare setting are effective in protecting HCP from COVID-19. These findings differ from reports describing known sources of exposures among HCPs in which healthcare exposures were the most reported (52.0%), followed by household exposure (30.8%) and community exposures (25.6%).^18^ These findings are not directly comparable to ours because these researchers included only known sources of exposure and during early stages of the pandemic (March 2020 to March 2021). Exposure trends were likely to change as the pandemic evolved. In the early stages of the pandemic, testing was scarce, and personal protective equipment was in limited supply, necessitating reuse. Limited community mobility reduced risk of community exposure, and incomplete mask use among HCP, along with changing quarantine isolation policies to support staffing shortages, might have contributed to increased risk of exposure in healthcare setting during the early stages of pandemic.

The COVID-19 rate among HCP correlated closely with COVID-19 community transmission level regardless of the source of exposure. This finding is not surprising because interventions that mitigate transmission in the community or household are only partially effective; multiple layers of mitigations are required to substantially reduce risk of infection.^19^ For example, social distancing was not consistently applied throughout the pandemic. Once infected, individuals transmit the virus whether they are asymptomatic, presymptomatic, or symptomatic.^20^ Individuals may unknowingly transmit or be exposed to infection even when they exercise caution and perceive their environment to be safe. This could explain why HCP were not able to identify their source of exposure in most cases. In addition, COVID-19 vaccines are only partially effective in preventing infection with emerging SARS-CoV-2 variants, further reducing its impact on transmission. Variance in testing capacity during early stages of pandemic also made identification and isolation of all infected individuals difficult, leading to inconsistent tracking and enforcement of infection prevention measures in communities and households.

We observed substantial variability of SARS-CoV-2 infection incidence among HCP. Registered nurses with unknown source of exposure had the highest incidence of 26.4%. Information about risk of exposure by hospital role is scarce. A meta-analysis conducted by He et al^21^ of SARS-CoV-2 antibodies among nurses reported seroprevalence comparable to other healthcare workers and possibly similar to the general population across different World Health Organization regions. In a cross-sectional sample of HCWs in a large metropolitan area, relative to physicians, the odds of SARS-CoV-2 infection for nurses were higher but not statistically significant (odds ratio, 2.33; 95% CI, 0.94–5.78).^22^ The specific factors that contributed to elevated risk among nurses remain unclear and should be the focus of future study. Our findings underscore the importance of infection prevention education for healthcare staff to address transmission through all source settings and not just limited to healthcare.

The strength of our study is its inclusion of a large HCP population with systematic identification and evaluation of new COVID-19 cases through a centralized process, along with the use of active surveillance from the onset of the pandemic through emergence and evolution of highly contagious SARS-CoV-2 BA.4, BA.5 o (omicron) variants. A standardized approach to case ascertainment and data collection minimized the risk of ascertainment, measurement, and recall bias. Our findings also fill a gap in our understanding of risk of infection among HCP with different hospital roles.

Among those with healthcare-associated infection, the proportion of 3 doses of vaccine was lower, indicating that booster vaccination may reduce the likelihood of infection in the healthcare setting even as SARS-CoV-2 variants emerge. The magnitude of differences in rate of infection by different sources of exposure is consistent with a known limited vaccine effectiveness in preventing transmission of infection. COVID-19 vaccine effectiveness was lower against infection, but estimated effectiveness against hospitalization and death remained high even during SARS-CoV-2 o (omicron) variant circulation. The protection also waned over time, especially against infection, and the risk of reinfection with the SARS-CoV-2 o (omicron) variant became appreciable 4 months following a booster.^23^ These findings emphasize the importance of both vaccination and boosters to protect oneself and others against the SARS-CoV-2 virus.

### Limitations

This study had several limitations. There was potential for measurement bias in our data because sources of infection in household, or community, could not be verified, whereas occupational exposures could more often be confirmed. Some unattributable infections may have been acquired through unrecognized occupational exposure. Similarly, it is possible that healthcare-associated cases were misclassified due to unknown exposure in the community. The strong correlation of infections with regional community infection rates across all categories of exposure suggests a possible effect of unrecognized community exposures among those classified as having healthcare-related exposure. However, our findings are consistent with a prior report from a large healthcare facility suggesting the validity of our findings.^10^ Because we included findings from an institution providing ambulatory and inpatient highest level of care, our findings may not be generalizable to all HCP across the United States.

### Future research

Future research should focus on the benefits of vaccination among HCP as new SARS-CoV-2 variants emerge. Ongoing and active surveillance of new SARS-CoV-2 infections among fully or partially vaccinated HCP and the level of protection is of interest because preserving the workforce is of paramount importance given the high ongoing burden of respiratory infections including COVID-19 on the strained healthcare systems.^24,25^

In conclusion, healthcare setting was not an important source of COVID-19 among our HCP. The majority of HCP were not able to identify the source of their SARS-CoV-2 exposure, followed by household and community exposures. HCP with community or unknown exposure were more likely to be unvaccinated.
